# Selenium Biofortification Differentially Affects Sulfur Metabolism and Accumulation of Phytochemicals in Two Rocket Species (*Eruca Sativa* Mill. and *Diplotaxis Tenuifolia*) Grown in Hydroponics

**DOI:** 10.3390/plants8030068

**Published:** 2019-03-16

**Authors:** Stefano Dall’Acqua, Andrea Ertani, Elizabeth A.H. Pilon-Smits, Marta Fabrega-Prats, Michela Schiavon

**Affiliations:** 1 Department of Pharmaceutical and Pharmacological Sciences, University of Padova, Via Marzolo 5, 35131 Padova, Italy; stefano.dallacqua@unipd.it; 2 DAFNAE, University of Padova, Agripolis, Viale dell’Università 16, 35020 Legnaro, Padova, Italy; andrea.ertani@unipd.it (A.E.); marta.fabrega87@gmail.com (M.F.-P.); 3 Department of Biology, Colorado State University, Fort Collins, CO 80523, USA; Elizabeth.Pilon-Smits@ColoState.EDU

**Keywords:** selenate, sulphur, glucosinolates, phenolics, amino acids, gene expression

## Abstract

Biofortification can be exploited to enrich plants in selenium (Se), an essential micronutrient for humans. Selenium as selenate was supplied to two rocket species, *Eruca sativa* Mill. (salad rocket) and *Diplotaxis tenuifolia* (wild rocket), at 0–40 μM in hydroponics and its effects on the content and profile of sulphur (S)-compounds and other phytochemicals was evaluated. *D. tenuifolia* accumulated more total Se and selenocysteine than *E. sativa*, concentrating up to ~300 mg Se kg^−1^ dry weight from 10–40 μM Se. To ensure a safe and adequate Se intake, 30 and 4 g fresh leaf material from *E. sativa* grown with 5 and 10–20 μM Se, respectively or 4 g from *D. tenuifolia* supplied with 5 μM Se was estimated to be optimal for consumption. Selenium supplementation at or above 10 μM differentially affected S metabolism in the two species in terms of the transcription of genes involved in S assimilation and S-compound accumulation. Also, amino acid content decreased with Se in *E. sativa* but increased in *D. tenuifolia* and the amount of phenolics was more reduced in *D. tenuifolia*. In conclusion, selenate application in hydroponics allowed Se enrichment of rocket. Furthermore, Se at low concentration (5 μM) did not significantly affect accumulation of phytochemicals and plant defence S-metabolites.

## 1. Introduction

The element selenium (Se) is essential in traces for ensuring human and animal health [1]. However, the boundary between Se deficiency and toxicity is extremely narrow for most organisms as compared to other micronutrients [1,2,3,4]. For humans, 55–70 μg Se is the recommended daily intake that guarantees adequate amounts of Se to be incorporated in the form of selenocysteine (SeCys) at the catalytic site of essential selenoproteins [5]. Selenium intake below 40 μg/day leads to deficiency [6] and may account for health-related disorders, like Keshan and Kashin–Beck diseases, hypothyroidism, reduced fertility and increased risk of infection and cancer development [1,7,8]. Conversely, chronic Se ingestion higher than the safe threshold can be responsible for a number of reported toxic symptoms [9,10].

To date, at least one billion people are estimated to experience Se deficiency [11]. This number is likely increasing due to the global climate-change, which is predicted to be associated with a dramatic reduction of soil Se content, especially in agricultural areas where soil Se is commonly lower than 2 ppm [12]. These soils are the most common worldwide [13] and areas with extremely low Se levels occur in parts of China, north western Europe and parts of eastern Europe, Southern Brazil and sub-Saharan Africa [14].

Plants are recognized as a major portal for Se entry into the food web and their Se content ultimately depends on soil Se. Thus, crops cultivated in soils with low Se levels normally contain negligible amounts of this element in their edible tissues. In this context, a number of fertilization strategies are broadly adopted to enrich plants in Se; from a human health perspective, it is important to pay attention to the Se concentration and chemical forms accumulated by plants [3,15]. Interestingly, plants do not have an essential requirement for Se [16,17,18,19] and most crops are non-accumulating species that cannot tolerate Se tissue concentrations higher than 10–100 μg g^−1^ dry weight [20]. However, species belonging to the Brassicaceae (Cruciferae) are typically good Se accumulators and also able to tolerate Se concentrations up to 1000 μg g^−1^ dry weight in their tissues. When biofortified with Se, these species can produce Se metabolites functioning as cancer-preventing agents, like Se-methylselenocysteine (SeMetCys) and γ-glutamyl-Se-methylselenocysteine [21,22,23,24].

Plants take up Se mainly as inorganic selenate or selenite, with selenate being more soluble and bioavailable under the typical oxidizing conditions of most soils. Selenate is chemically similar to sulphate, therefore it enters the root cells and moves throughout the plant using high- and low-affinity sulphate transporters (SULTR) [25,26,27]. Once inside cells, selenate enters the plastids and accesses the sulphur (S) metabolic pathway. First, it is activated by the enzyme ATP sulfurylase (ATPS) to form adenosine 5′-phosphoselenate (ATPSe). Then, activated selenate is reduced via selenite to selenide and assimilated into SeCys and selenomethionine (SeMet). Se-amino acids can replace their S-analogues, amino acids cysteine (Cys) and methionine (Met) in proteins, causing disruption of their folding and function and thus impairing cell metabolism [28,29,30].

Because plants cannot discriminate between Se and S during their transport and assimilation, Se biofortification in crops is strongly influenced by the concentration of the competitor sulphate. On the other hand, increased accumulation of Se in plants might affect the synthesis of S-related compounds involved in the plant’s defences against stress, such as glutathione (GSH) and glucosinolates (GLS). Glucosinolates are almost exclusively found in Brassicaceae and are nitrogen (N)- and S-containing glycosides that give plants protection from insect and herbivore predators [31,32]. It has been reported that Se at high concentration may induce oxidative stress in plants via reduction of intracellular GSH due to cysteine depletion [29,30], while contrasting results exist about the effects of Se fertilization on GLS content, likely because of different Se application protocols and the plant species used for Se enrichment [33,34,35,36]. Also, the method of Se application may be a crucial factor influencing GLS accumulation in different plant organs [37,38].

Selenium fertilization may alter the synthesis of additional metabolites with recognized nutritional properties, like phenolic acids and amino acids [37,39,40], which are particularly abundant in cruciferous vegetables. Both phenolics and amino acids are N-compounds and Se may affect N metabolism via either interaction with the S pathway and/or reduction of molybdenum (Mo) uptake, Mo being an indispensable cofactor for the activity of nitrate reductase [37,40].

Taking into account all of the above, Se biofortification efforts should consider the concentration and species of Se accumulated in the plant in relation to the effects that such Se enrichment could exert on the production of health-beneficial and/or stress-defence compounds. Therefore, in the current study we attempted to (i) biofortify with Se two rocket species, *Eruca sativa* and *Diplotaxis tenuifolia* (Brassicaceae), through application of different selenate concentrations in a hydroponic set-up and highlight differences in their Se and S accumulation capacity, (ii) evaluate the effects of plant Se concentrations on the content of GLS and other important phytochemicals (GSH, phenolics and amino acids). *Diplotaxis tenuifolia* is a wild relative of the crop species *Eruca sativa*. Both species are edible and differ in profile of phytochemicals [41].

Furthermore, to get a better insight on the impact of Se fertilization on S and GLS accumulation in these cruciferous vegetables, the effect of selenate application was investigated at the molecular level by analysing the expression of genes involved in sulphate/selenate transport (SULTR), S/Se-assimilation (ATP sulfurylase (ATPS) isoforms), GLS biosynthesis (branched-chain aminotransferase 3 (BACT3), methyl-thioalkyl-malate synthase 1 (MAM1), UDP-glucose:thiohydroximic acid S-glucosyltransferase (UGT74B1) and GLS regulation (MYB28) and breakdown (myrosinase, MYR).

## 2. Results

### 2.1. Effect of Selenium on Plant Growth, Se and S Accumulation

**Figure 1:**
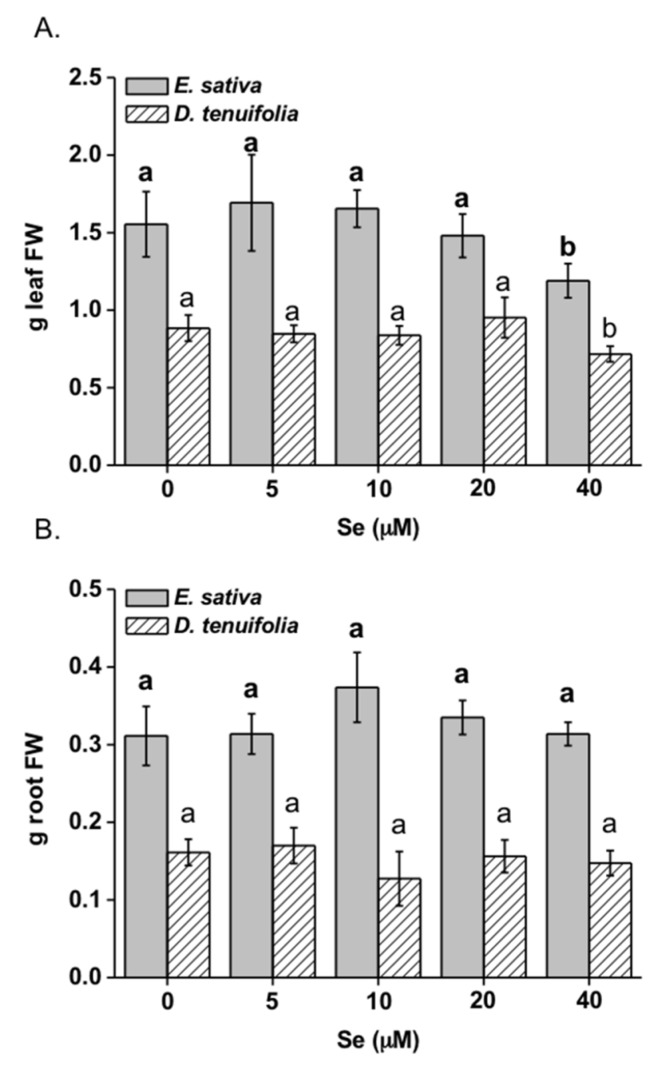
Fresh weight (FW) of leaves (**A**) and roots (**B**) of *E. sativa* and *D. tenuifolia* plants grown in hydroponics with 0–40 μM selenate. The FW reported is the average FW of each leaf (±SD, *n* = 9). Different letters in bold above bars indicate significant differences between the means (*p* < 0.05) of values referred to *E. sativa*, while different letters not bolded indicate significant differences between the means (*p* < 0.05) of values referred to *D. tenuifolia*.

The application of Se concentrations from 5–20 µM did not affect the leaf fresh weight of the two rocket species (Figure 1A, Figure S2). However, Se applied at higher concentration (40 μM) was associated with a decline in their leaf biomass (by 23% and 18% for *E. sativa* and *D. tenuifolia*, respectively). The root fresh weight was not altered by increasing Se concentrations (Figure 1B, Figure S2). The leaf and root dry weight showed the same trend as the fresh weight (data not shown).

**Figure 2:**
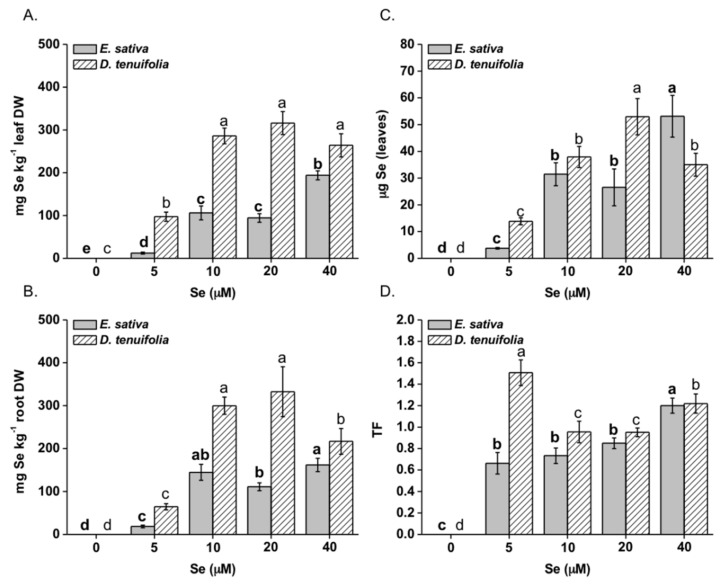
Total Se concentration in leaves (**A**) and roots (**B**) of *E. sativa* and *D. tenuifolia* plants grown in hydroponics with 0–40 μM selenate. Content of total Se per plant (relatively to the leaf edible part) (**C**) and translocation factor, TF (calculated as the Se shoot:Se root ratio) (**D**). Data shown are the mean ± SD of three replicates. Different letters in bold above bars indicate significant differences between the means (*p* < 0.05) of values referred to *E. sativa*, while different letters not bolded indicate significant differences between the means (*p* < 0.05) of values referred to *D. tenuifolia*.

The two species exhibited strong differences in their capacity to take up Se, with *E. sativa* accumulating less Se than *D. tenuifolia* (Figure 2A,B). Leaf Se concentration in *E. sativa* was highly correlated with Se in the nutrient solution (R^2^ = 0.91), attaining the maximum value (~200 mg Se kg^−1^ DW) when plants were treated with 40 μM Se (Figure 2A). Under Se concentrations equal to 10 or 20 μM Se, this value was reduced by about 2-fold. Conversely, leaf Se accumulation in *D. tenuifolia* reached a plateau of ~300 mg Se kg^−1^ DW when supplied with Se concentrations at/above 10 μM. In roots, the trend of Se accumulation for both species was the same as in leaves up to 20 μM Se but at higher Se concentration (40 μM) no further increase in Se enrichment was obtained in *E. sativa*, while a decrease was evident in *D. tenuifolia* (Figure 2B). Calculating the total μg Se per plant on average (considering only the edible leaf portion), values were higher in *D. tenuifolia* than in *E. sativa* plants treated with 5–20 μM Se (Figure 2C). However, *E. sativa* accumulated more Se when plants were grown with 40 μM Se. The Se root-to-shoot translocation (TF) values were overall close to 1 or higher (Figure 2D).

**Figure 3:**
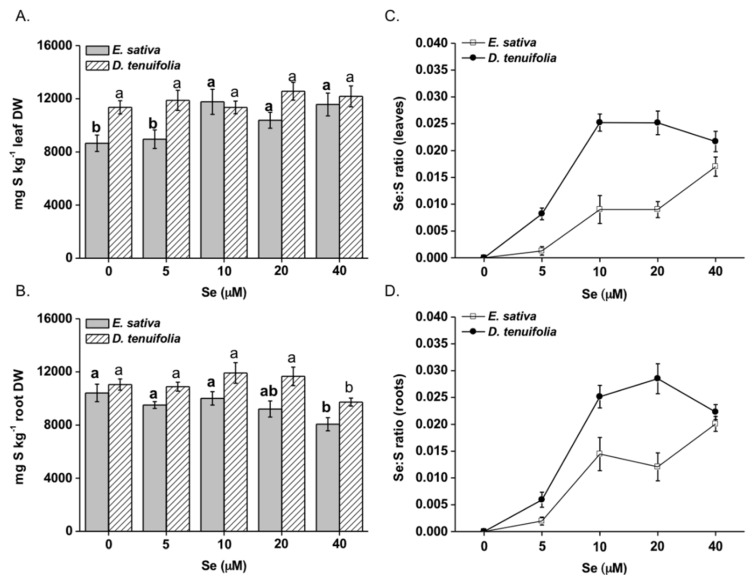
Total S concentration in leaves (**A**) and roots (**B**) of *E. sativa* and *D. tenuifolia* plants grown in hydroponics with 0–40 μM selenate. Data shown are the mean ± SD of three replicates. Different letters in bold above bars indicate significant differences between the means (*p* < 0.05) of values referred to *E. sativa*, while different letters not bolded indicate significant differences between the means (*p* < 0.05) of values referred to *D. tenuifolia*. Se:S ratio in leaves (**C**) and roots (**D**) of the two species.

In general, *E. sativa* contained less S than *D. tenuifolia* (Figure 3A,B) but the leaf S concentration increased with increasing Se concentrations (Figure 3A). An opposite trend was observed for roots, in which S concentration appreciably decreased, especially when plants were supplied with 20 or 40 μM Se (Figure 2D). In *D. tenuifolia*, leaf S concentration was not affected by Se treatment but a reduction of S accumulation happened in the roots of plants treated with 40 μM Se. The leaf and root Se:S ratios were significantly greater in *D. tenuifolia* than in *E. sativa*, within the 5–20 μM Se range (Figure 3C,D). However, these differences were not apparent when plants were supplied with 40 μM Se.

### 2.2. Effect of Se on Plant S Compounds

**Figure 4:**
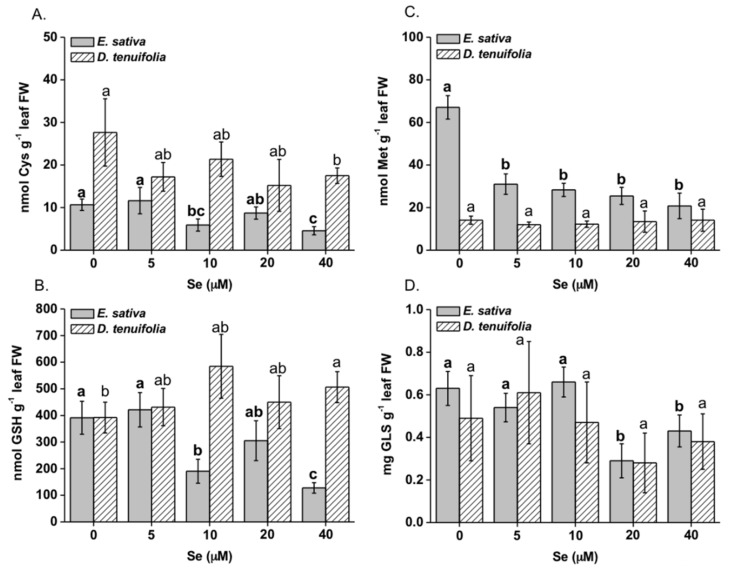
Content of cysteine (Cys, **A**), total glutathione (GSH, **B**), methionine (Met, **C**) and glucosinolates (GLS, **D**) in leaves of *E. sativa* and *D. tenuifolia* plants grown in hydroponics with 0–40 μM selenate. Data shown are the mean ± SD of three replicates. Different letters in bold above bars indicate significant differences between the means (*p* < 0.05) of values referred to *E. sativa*, while different letters not bolded indicate significant differences between the means (*p* < 0.05) of values referred to *D. tenuifolia*.

Selenium treatment higher than 5 μM resulted in the reduction of cysteine (Cys) content in leaves of *E. sativa* plants, with values about 2 fold lower than those measured in control plants (Figure 4A). Glutathione (GSH), of which cysteine is the precursor, showed the same trend toward reduction (Figure 4B), while methionine (Met) accumulation was dramatically reduced by all Se treatments, that is, from 2- to 4-fold compared to the untreated plants (Figure 4C). In *D. tenuifolia*, the leaf Cys content was reduced only by the 40 μM Se treatment (Figure 4A) under which, conversely, GSH increased (Figure 4B). Methionine content was overall unchanged in this species (Figure 4C).

With respect to total glucosinolates (GLS), a decrease in their content was observed in leaves of both species on treatment with Se (Figure 4D). However, the effect was more pronounced in *E. sativa* (reduction by about 30–50%) and was not significant in *D. tenuifolia*. The individual GLS compounds identified in the rocket species are reported in Table 1 and Table S1. They included glucoraphanin, glucocheirolin, glucoerucin, dimeric-4-mercaptobutyl glucosinolate (DMB-GLS), glucosativin, neoglucobrassicin. Their identification was performed by comparison of fragmentation spectra with those reported in the literature, that is, Griffiths et al. [42] for glucoraphanin, Pasini et al. [41] for DMB-GLS and glucosativin, Matthäus et al. [43] for glucocheirolin, Kusznierewicz et al. [44] for glucoerucin and neoglucobrassicin. Among them, the most abundant were glucoraphanin and DMB-GLS, followed by glucoerucin and glucosativin, the last GLS being high only in *E. sativa* on Se treatments greater than 5 µM (Table 1). In a previous study however, glucosativin was found to be prominent [41]. This was probably because we may have used a different *E. sativa* accession or due to the protocol used for extraction and quantification. The growth condition could also influence the relative content of GLS. A reduction in glucoraphanin, glucoerucin and DMB-GLS was observed in *E. sativa*. In *D. tenuifolia*, there was a big variation in the content of glucoraphanin and DMB-GLS among plants of the same treatment but their decline in response to increasing Se concentration was overall not significant (Table 1). We did not detect the presence of selenoglucosinolates (Se-GLS), which have been previously detected in other Brassicaceae spp. [45].

### 2.3. Effect of Se on S Transport and Assimilation Genes

**Figure 5:**
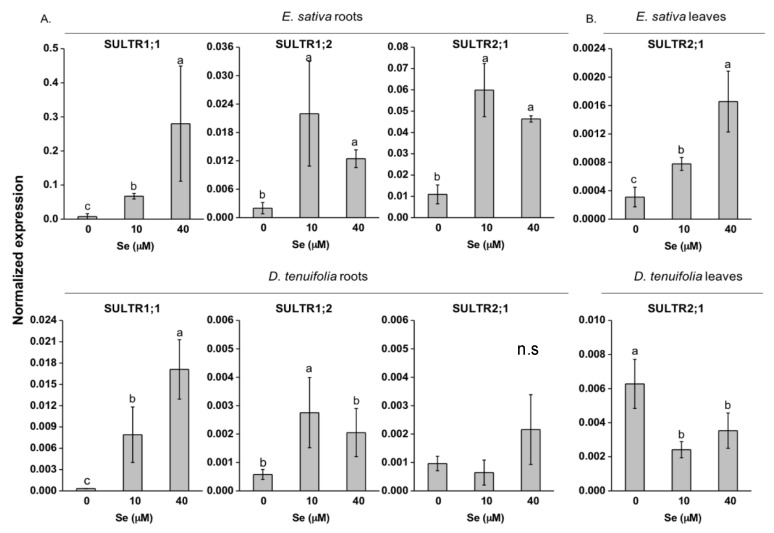
Expression profiling by real-time RT-PCR of sulphate transporter genes coding for SULTR1;1, SULTR1;2 and SULTR2;1 in roots (**A**) and SULTR2;1 in leaves (**B**) of *E. sativa* and *D. tenuifolia* plants grown in hydroponics with 0–40 μM selenate. Data shown are the mean ± SD of three replicates. Different letters above bars indicate significant differences between the means (*p* < 0.05). n.s. = not significant differences between means.

Application of Se to rocket plants led to significant changes in the transcript abundance of root high affinity (SULTR1) and low affinity (SULTR2) sulphate transporter genes (Figure 5A,B). A clear Se dose-dependent increase was observed for SULTR1;1 expression in the two species, while SULTR1;2 transcription was up-regulated to similar levels by 10 and 40 μM Se (Figure 5A). The trend of SULTR2;1 transcript accumulation in *E. sativa* roots was the same as SULTR1;2, while a weak SULTR2;1 up-regulation in *D. tenuifolia* roots was observed under high Se (40 µM) treatment (Figure 5A). In *E. sativa* leaves, the expression of the low-affinity sulphate transporter SULTR2;1 was increasingly Se-dose dependent (Figure 5B). In contrast, SULTR2;1 transcription in *D. tenuifolia* leaves was appreciably reduced by both Se concentrations (Figure 5B).

**Figure 6:**
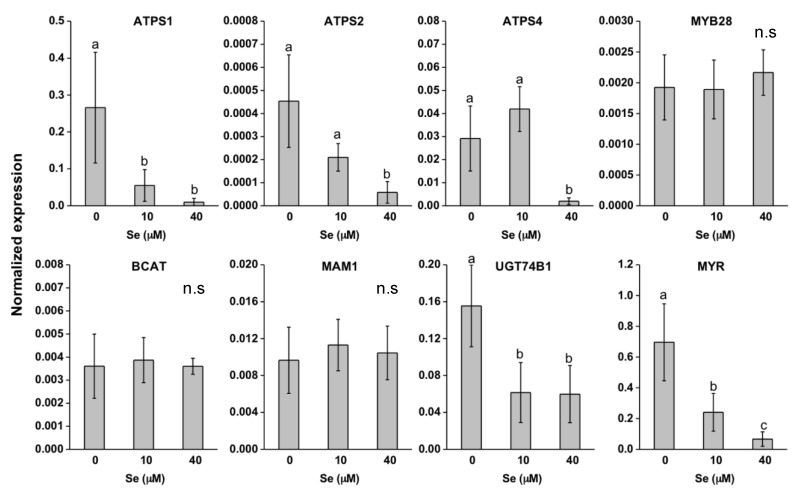
Expression profiling by real-time RT-PCR of genes coding for ATP sulfurylase isoforms (ATPS1, ATPS2, ATPS4), MYB28, BCAT, MAM1, UGT74b1, MYR in leaves of *E. sativa* plants grown in hydroponics with 0–40 μM selenate. Data shown are the mean ± SD of three replicates. Different letters above bars indicate significant differences between the means (*p* < 0.05). n.s. = not significant differences between means.

**Figure 7:**
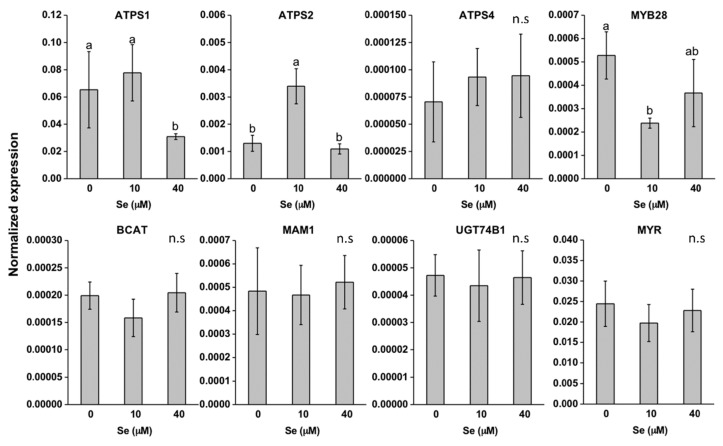
Expression profiling by real-time RT-PCR of genes coding for ATP sulfurylase isoforms (ATPS1, ATPS2, ATPS4), MYB28, BCAT, MAM1, UGT74b1, MYR in leaves of *D. tenuifolia* plants grown in hydroponics with 0–40 μM selenate. Data shown are the mean ± SD of three replicates. Different letters above bars indicate significant differences between the means (*p* < 0.05). n.s. = not significant differences between means.

With respect to ATP-sulfurylase (ATPS) isoforms, that is the enzymes involved in the first step of Se/S assimilation, the ATPS1 gene was much more expressed than ATPS2 and ATPS4 genes in both species (Figure 6 and Figure 7). In *E. sativa* leaves, all genes coding for ATPS isoforms were down-regulated by Se and this effect was more pronounced for ATPS1 (Figure 6). Expression of ATPS1 was repressed in *D. tenuifolia* leaves too but only when plants were treated with 40 μM Se (Figure 7). The genes coding for other ATPS isoforms in this species showed a different pattern of regulation in response to Se treatment: ATPS4 and ATPS2 transcript abundance were unaffected and more expressed, respectively, under low Se than the control treatment.

To evaluate the effect of Se on GLS production, the transcript levels were analysed of a number of genes involved in the synthesis and break-down of these compounds (Figure 6 and Figure 7). Expression of MYB28, BCAT and MAM1 was unchanged by Se in *E. sativa* leaves, while UGT74B1 and MYR transcripts were significantly down-regulated (Figure 6). Overall, expression of all GLS-related genes in *D. tenuifolia* was not altered by Se, with the exception of MYB28, whose transcription was repressed under low Se (Figure 7).

### 2.4. Effect of Se on Amino Acids and Phenolic Compounds

**Figure 8:**
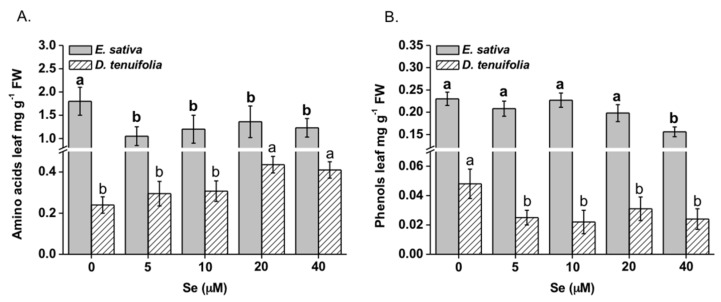
Content of total amino acids (**A**) and phenolics (**B**) in leaves of *E. sativa* and *D. tenuifolia* plants grown in hydroponics with 0–40 μM Se. Data are the mean ± SD of three replicates. Different letters in bold above bars indicate significant differences between the means (*p* < 0.05) of values referred to *E. sativa*, while different letters not bolded indicate significant differences between the means (*p* < 0.05) of values referred to *D. tenuifolia*.

Different trends were observed in the two rocket species with respect to Se-dependent accumulation of amino acids (Figure 8A). Specifically, all Se concentrations reduced the content of amino acids in *E. sativa* (by about 25–45%) and conversely, they induced a Se dose-depended increase (R^2^ = 0.68) of these metabolites in *D. tenuifolia*.

Amino acids whose content was significantly reduced by Se in *E. sativa* included proline, glutamine, phenylalanine and tyrosine. The same amino acids, except phenylalanine, were more accumulated in *D. tenuifolia* after Se application (Table 2). Proline content was increased by about 10-fold in plants of this species treated with 20 and 40 μM Se compared to control plants. Among Se amino acids, only SeCys was detected and its content in *D. tenuifolia* was about 2.5-fold higher than in *E. sativa*.

Variation in individual polyphenols identified in leaves of *E. sativa* and *D. tenuifolia* is reported in Table 3. Results indicate that they mainly consisted of flavonoid derivatives. In particular, glycosylated derivatives of kaempferol and isorhamnetin, often esterified with phenylpropanoid acids, were prevalent in *E. sativa*. On the other hand, derivatives of quercetin and kaempferol were the most abundant in *D. tenuifolia*. Some compounds were detected in both species, like kaempferol-3,4’-diglucoside, isorhamnetin-3,4-diglucoside, quercetin-3-glucoside, quercetin-3,4’-diglucoside. These findings are in agreement with previous work concerning phenol compounds identification in rocket species [41,46,47].

Overall, the total content of phenols significantly decreased (by about 27%) in *E. sativa* plants supplied with 40 µM Se (Figure 8B). A similar decline (by about 33–50%) was observed in *D. tenuifolia* plants treated with Se, regardless of the dose applied (Figure 8B). Such reductions were mainly due to the decline in content of the prevalent phenol compounds recorded in untreated plants of the two species, that is kaempferol-3,4’-diglucoside, kaempferol-3-glucoside and kaempferol-3-(2-sinapoil-glucoside)-4’-glucoside in *E. sativa*, quercetin-3,4’-diglucoside, quercetin-3,3,4’-triglucoside and kaempferol-3.4’-diglucoside in *D. tenuifolia* (Table 3).

## 3. Discussion

Biofortification is a current strategy exploited to improve the plant content in beneficial elements and molecules with valuable properties for consumers. In this study, we tested the effects of different Se concentrations on the capacity of two rocket species to accumulate Se and assayed their impact on the synthesis of S compounds. Selenium and S can compete for the same uptake and assimilation pathways in plants and thus Se enrichment may affect the production of S-containing metabolites [33,34,35,36,37,48]. Furthermore, as Se is known to influence nitrogen primary and secondary metabolism in plants, we evaluated the variation in content of amino acids and phenolic compounds in response to Se application.

Our results highlight significant differences in Se accumulation between *E. sativa* and *D. tenuifolia*, with both species showing high potential for Se root-to-shoot transfer and tolerance (Figure 2D). It must be noticed that *E. sativa* species has been artificially selected and this fact could have been responsible for some of such differences. With respect to *E. sativa* plants, Se concentrations within the range 5–20 μM look promising in order to provide a daily amount of Se equal to 70 μg, which is the maximum amount recommended for adults [5]. In this case, the consumption of 30 g plant fresh material derived from *E. sativa* plants grown with 5 μM or 4 g plant fresh material obtained from the same species but grown with either 10 or 20 μM Se, would meet the daily consumers’ requirement for Se. On the other side, *D. tenuifolia* showed greater capacity than *E. sativa* to accumulate Se under low Se (5 μM) treatment, thus consumers could carefully feed on about 4 g plant fresh material only from *D. tenuifolia*. As this amount is very small, *D. tenuifolia* fresh leaves could be more properly added in mixed salads. Application of Se concentrations equal or higher than 10 μM caused Se accumulation in *D. tenuifolia* likely too high to ensure a safe daily Se intake (on a fresh weight basis). Therefore, lower Se dosages should be applied to this species.

Selenocysteine (SeCys) was the sole Se amino acid retrieved in rocket and variation in its content between the two species reflected differences in their capacity to accumulate Se (Table 2). The SeCys concentration in *D. tenuifolia* was 2.5-fold higher than in *E. sativa*. With these levels, consumption of 50 g leaves of *E. sativa* plants treated with 5 μM Se could ensure amounts of organic Se (64 μg) in the diet that approach the recommended daily Se intake. Feeding on leaves derived from either *E. sativa* grown with Se 10–20 μM or *D. tenuifolia* supplied with Se 5 μM would provide less SeCys to consumers, that is about 23 and 48 μg, respectively, because of the smaller quantity of plant material recommended for safe consumption based on its total Se content.

Differences in Se and SeCys contents between *E. sativa* and *D. tenuifolia* possibly suggest the existence of distinct regulatory mechanisms of Se/S uptake and assimilation in response to Se exposure, which could be in part ascribed to different levels in S (generally higher in *D. tenuifolia*) and properties of sulphate transporters (SULTR) and assimilation enzymes (Figure 5, Figure 6 and Figure 7). The expression of root high affinity selenate/sulphate transporters (SULTR1) involved in primary Se/S uptake was induced by Se in both *E. sativa* and *D. tenuifolia* plants. This effect was previously observed in other Brassicaceae species [25,26,37,49] but in our study it did not correspond with increased S accumulation. *D. tenuifolia* contained more S and Se in tissues than *E. sativa*, perhaps because of greater capacity of SULTR1 permeases for S and Se transport across the root plasma membranes.

*E. sativa* plants showed enhanced re-mobilization of S in response to increasing Se application, which was likely dependent upon SULTR2;1 up-regulation (Figure 5). This transporter is expressed in the xylem and phloem parenchyma cells of leaves and xylem parenchyma and pericycle cells of roots in *Arabidopsis thaliana* and mediates the movement of sulphate into the vascular bundle [50]. Therefore, SULTR2;1 has an established role in Se/S root to shoot translocation [51]. Despite higher SULTR2;1 expression and S translocation to the shoot, *E. sativa* plants contained less Cys and GSH in leaves when treated with Se concentrations equal or higher than 10 μM (Figure 4A,B), which could be explained by low expression of ATPS genes and perhaps other enzymes implied in Cys biosynthesis [48]. Also, because SeCys accumulation increased at high Se dosages in *E. sativa*, it is conceivable that decreased accumulation of S-compounds could be in part ascribed to the interference of Se with the S flow through the assimilation pathway. Consistent with our findings, reduction in thiols (Cys and GSH) and down-regulation of ATPS1 gene, which encodes a major isoform rate-limiting for Se/S assimilation [24,52], were previously observed in leaves of radish plants treated with Se [37]. In contrast, high S levels in *D. tenuifolia* perhaps helped plants in maintaining Cys and GSH contents under Se concentrations within 5–20 μM. Reduction in Cys and in S but not in GSH, was observed when plants were treated with 40 μM Se (Figure 4A,B). This result suggests that, in addition to Se/S competition for the synthesis of Se/S amino acids, higher consumption of Cys may occur in *D. tenuifolia* plants to sustain a steady level of GSH to act against oxidative stress, likely generated by high Se accumulation occurring at 40 μM Se. GSH is indeed known for its involvement in plant defence against a variety of abiotic stresses, including Se-induced toxicity [29,30]. It is noteworthy that transcription of SULTR2;1 in *D. tenuifolia* roots was not significantly affected by Se, while it slightly decreased in leaves. Possibly, as a defence mechanism, this species tried to prevent further translocation of Se to the aerial tissues where Se concentration was already elevated, according to the observation that Se accumulation in leaves achieved a plateau when Se concentrations were in the range of 10-40 μM (Figure 2A,B).

Selenium application reduced the synthesis of total GLS in *E. sativa*, which mainly consisted of aliphatic GLS derived from methionine (Table 1). Cysteine is the precursor of methionine; thus we can postulate that the decrease in content of both amino acids may justify the observed decline in aliphatic GLS in this species. In support of our hypothesis, recent work performed in other Brassicaceae spp. has shown that reduction of GLS in response to Se application is due to negative effects on the production of precursor amino acids and genes involved in GLS biosynthesis [37,48]. Accordingly, in *E. sativa* leaves a repression in transcription of some genes involved in the synthesis (UGT74B1) and hydrolysis (MYR) of GLS was revealed. Conversely, in *D. tenuifolia* methionine and GLS content did not show significant variation, nor did the expression of several GLS-related genes (BCAT, MAM1, UGT74b1 and MYR). These findings enforce the assumption that Se did not exert substantial effects on S assimilation in this species. In this respect, a number of studies exist that show contrasting results on the effects of Se on the production of GLS in cruciferous vegetables [33,36,37,48,53,54,55]. These differences are also probably related to the method of Se application to plants, chemical form and concentration of Se applied, number of Se applications, plant species subjected to biofortification and developmental stage [38,55,56]. Hsu et al. [54] and McKenzie et al. [55] for instance, did not observe a reduction in GLS content in broccoli when plants were fertilized with Se and this was likely because they applied a more moderate amount of Se to plants.

The reduction in content of GLS observed in *E. sativa* plants might have potential ecological implications for this species. GLS are indeed notorious compounds implied in plant defences against herbivore predators and pathogens and their decrease could affect the plant capacity to prevent damages caused by these organisms, which could result in loss of crop yield.

Differential effects of Se on other compounds with recognized nutritional properties, that is amino acids and phenolics, were observed in the two rocket species (Table 2 and Table 3). Previous work showed that the content of such compounds could be affected by Se [37,39]. In this study, Se altered the content of several amino acids in both species. Specifically, Se strongly promoted proline accumulation in *D. tenuifolia*. This amino acid functions as osmolyte in cells and therefore its increase could be intended as a defence response against osmotic and oxidative stress likely caused by high Se in this species. Variation in proline content was previously reported in Se-enriched radish [37] and in soybean [57] plants. In contrast, Se exerted a negative effect on the synthesis of proline and other amino acids (glutamine, phenylalanine and tyrosine) in *E. sativa*. It is noteworthy however, that this species contained much higher levels of these amino acids than *D. tenuifolia*, regardless of Se supplementation.

Accumulation of phenolic compounds was depleted in *D. tenuifolia* upon all Se concentrations applied, while a decrease in their content was manifest in *E. sativa* only when plants were treated with high Se concentration. Similar results were previously reported by Tian et al. [36], while the increase of phenolic acids, mainly in their soluble conjugated forms, was observed in rice by D’Amato et al. [58]. Such contrasting results likely were due to different methodological approaches (foliar fertilization or soil amendment) used for Se enrichment of plants, as well as to the form of Se applied (mainly selenite or selenate). Phenylalanine beyond being the precursor of phenolic compounds, functions as a substrate for aromatic GLS. Although we have not detected phenylalanine-derived GLS in rocket, their occurrence has been reported in this species [59,60,61]. The lack of correlation between phenylalanine and total phenolic compounds in both species leaves opens the hypothesis that Se could interfere with the shikimate pathway in multiple ways, that is by diverting phenylalanine from being used for phenol biosynthesis to serve as a substrate for GLS generation, and/or via direct inhibition of one or more enzymes involved in the pathway.

## 4. Materials and Methods

### 4.1. Experimental Set Up

Rocket (*Eruca sativa* and *Diplotaxis tenuifolia*, Corona sementi, Mortegliano, UD) seeds were surface-sterilized as described by Schiavon et al. [37] and then kept to germinate and grow for 8 d in half-strength MS agar medium [62] inside a growth chamber under the following conditions: 14 h light/10 h dark cycle, 26/21 °C air temperature, relative humidity of 70/85% and at a photon flux density (PFD) of 280 mol m^−2^s^−1^.

Seedlings were then shifted to 3 L pots holding a nutrient solution with this composition (mM): KH_2_PO_4_ (0.63), Ca(NO_3_)_2_ (2), KNO_3_ (3), MgSO_4_ (1.5), FeNaEDTA (0.040), plus micronutrients. The nutrient solution was thoroughly aerated and changed every 3 days. At 30 days after transplant, Se as selenate (Na_2_SeO_4_) was applied to the nutrient solution at 5, 10, 20 or 40 μM. These concentrations were chosen based on preliminary experiments where the two rocket species were grown in MS agar medium (Figure S1). A group of plants that was not supplied with selenate served as control. For each experimental condition, five pots were prepared, with a density of 6 plants per pot. The experimental design for plant growth was randomized (the pots were re-arranged twice a week).

Plants were harvested one week from the starting of the Se treatment, washed with distilled water and dried with blotting paper. Leaves and roots of remaining plants were frozen in liquid nitrogen and kept at −80 °C for further analyses. Nine plants per treatment (3 per pot) were divided into roots and shoots and their fresh weight was measured individually. Plant material was then placed inside a drying oven for 2 d at 70 °C to measure their dry weight.

### 4.2. Determination of Total Se and S

Leaf and root tissues of rocket plants were dried for 48 h at 80 °C and further digested using nitric acid according to the method reported by Zarcinas et al. [63]. Inductively coupled plasma atomic emission spectroscopy (ICP-AES) was used according to the protocol by Fassel [64] to determine each digest’s Se and S elemental concentrations. Analyses were conducted in triplicates (1 replicate = 1 plant).

### 4.3. Identification and Quantification of Glucosinolates

Extraction of glucosinolate from rocket leaves was conducted using a modified protocol by [65]. To prevent myrosinase activity in the samples, glucosinolates were extracted from 6 g of leaves boiled for 4 min in 18 mL of a methanol/water solution (ratio 70:30, *v*/*v*). Sinigrin (1.26 mg/mL) was added as internal standard to this solution. In order to achieve the complete extraction of glucosinolates, leaf material residual after sample filtration was re-extracted using 70% (*v*/*v*) methanol for 4 min. The two extracts from each sample were further combined and purified through a SPE (Solid-Phase Extraction) column (0.8 × 4 cm, Agilent Technologies) equipped with 0.256 g of an ion-exchange resin (DEAE-SEPHADEX-A25) imbibed in 4 mL of a 0.5 M Na-acetate buffer solution (pH = 5). The column was initially washed with 1 mL deionized H_2_O and then loaded with 2.5 mL extract containing the internal standard. The further purification steps were performed according to the protocol reported by Schiavon et al. [37].

The analysis of glucosinolates was performed in HPLC-MS and the Electrospray Ionization (ESI) as a source in the full scan positive ion-mode. The analysis of the fragmentation patterns of spectra shown in Table S1 was realized through the Turbo Detection Data Scanning (TDDS) function. The chromatographic separation was performed using a column Eclipse XDB C-8 5 μm 2.1 × 150 mm as described by Schiavon et al. [37]. For the quantification of glucosinolates, glucoerucin was used as reference standard at different concentration levels. Analyses were performed on three biological replicates (1 replicate = 1 plant).

### 4.4. Identification and Quantification of Polyphenols

Extraction of polyphenols from three replicates of frozen rocket tissues was performed using a methanol:water (1:1, *v*/*v*) solution in ultrasonic bath for 15 min. The ratio of plant material to mixture was 1:10 (*w*/*v*) and extracts were filtered at 0.45 μm (Millipore, Burlington, MA, USA). Validation of the extraction procedure was realized by measuring the recovery percentage of chlorogenic acid and rutin in replicates of leaf samples.

Qualitative and quantitative analyses of polyphenols were realized both via HPLC-MS and HPLC-DAD. For the separation of polyphenols, an Eclipse Plus C-18 column (3.5 μm × 2.1 mm × 150 mm, Agilent, Santa Clara, CA, USA) was used in HPLC system Varian 212 at 35 °C as reported in Schiavon et al. [37].

The identification and quantification of the principal polyphenols in the extracts was conducted via Ion Trap Mass Spectrometry (Varian 500 MS, Palo Alto, CA, USA) coupled to the HPLC system, by comparison with appropriate standards (chlorogenic acid for phenols, rutin for flavonoids) and analysis of the fragmentation patterns of spectra (Table S2) through the Turbo Detection Data Scanning (TDDS) function. Electrospray Ionization (ESI) was used as source in negative ion-mode and the mass range considered was within 50–3500 uma. Each sample’s volume injected was equal to 10 μL.

### 4.5. Free Amino Acids

Extraction of free amino acids, including Se-amino acids, were obtained from three replicates of frozen rocket leaves (500 mg) using 0.1 M HCl (1:4 (*w*/*v*). The extracts underwent centrifugation at 4 °C for 10 min at 10,000 rpm. The supernatants were collected and filtered at 0.45 μm (Millipore). Qualitative and quantitative analyses of amino acids were realized through HPLC-MS equipped with a ZORBAX Eclipse Plus AAA column (3.5 μm × 3 × 150 mm) as described by Schiavon et al. [37].

The identification and quantification of the amino acids in the extracts was attained via Ion Trap Mass Spectrometry (Varian 500 MS) coupled to the HPLC system, by comparison with appropriate standards and analysis of the fragmentation patterns of spectra (data not shown) through the Turbo Detection Data Scanning (TDDS) function [37]. For the identification and quantification of the amino acids the reference standards consisted of these amino acids: Alanine, Arginine, Asparagine, Aspartic acid, Cysteine, Glutamine, Glutamic acid, Glycine, Histidine, Isoleucine, Leucine, Lysine, Methionine, Phenylalanine, Proline, Serine, Threonine, Tryptophan, Tyrosine, Valine, Selenomethionine, Selenocysteine, Se-Methyl-Selenocysteine.

### 4.6. Determination of Low Molecular Weight Thiol Compounds

Frozen leaf material (250 mg) from were ground in liquid nitrogen with 0.1 N HCl and 1 mM EDTA. Extracts were centrifuged at 10,000 g for 10 min and then analysed for low-molecular-weight (LMW) thiol contents. Extracts (50 μL) were further derivatized using SBD-F fluorophore (Sigma-Aldrich, St. Louis, MO, USA). Low-molecular-weight thiols (cysteine and total glutathione) were separated by isocratic HPLC according to Masi et al. [66]. The mobile phase was 3% methanol in 75 mM NH_4_^+^ formiate, pH 2.9.

### 4.7. Gene Expression via qRT-PCR

For quantitative Real-Time PCR experiments, RNA was extracted from three individual samples (100 mg) of roots and leaves of rocket plants cultivated in hydroponics in the presence of 0 Se (control), 10 μM Se, 40 μM Se. RNA was extracted using a phenol/chloroform protocol [67]. All the cDNAs were synthesized from 3 μg of RNA using 200 U of ImProm-II™ Reverse Transcriptase (Promega, Milano, Italy) and oligodT as primers (reaction volume = 20 μL) as described in Schiavon et al. [37]. Specific primer pairs were designed based on conserved sequences selected among *Brassicaceae* spp. (Table S3). Gene expression analyses were conducted as described by Schiavon et al. [37], using a thermal cycler 7300 Real-Time PCR System (Applied Biosystem). Each qPCR reaction (10 μL final volume) contained 1 μL of 1:10 diluted cDNA, 1 μL of primer couple (10 μM) and 5 μL of 2× SYBR Green PCR Master Mix according to the manufacturer’s instructions. The thermal cycling conditions were used: 95 °C for 10 min, 50 cycles of 95 °C for 15 sec, 60 °C for 1 min). The gene expression analysis for each biological replicate was performed in two technical replicates (only one set of data is reported in figures).

All quantifications were normalized to the housekeeping actin gene. The CT values were analysed with the Q-gene software by averaging three independently calculated normalized expression values for each sample. Expression values are given as the mean of the normalized expression values of the triplicates, calculated according to Equation (2) of the Q-gene software [68].

### 4.8. Statistical Analysis

Analysis of variance (ANOVA) was performed using the SPSS software and was followed by pair-wise post-hoc analyses (Student-Newman-Keuls test) to determine which means differed significantly at *p* < 0.05 (±SD).

## 5. Conclusions

In conclusion, application of selenate to *D. tenuifolia* and *E. sativa* plants growing in hydroponics proved to be successful in Se biofortification attempts of both rocket species. Low Se concentration, especially, did not significantly affect the content of most phytochemicals analysed. Differences in Se accumulation were observed between the two species and likely were due to their different S content and ability to either maintain or increase the level of molecules (e.g., GSH, proline) that function in reactive oxygen species (ROS) scavenging under oxidative and osmotic stress induced by high Se. The elevated S content in *D. tenuifolia* appeared to prevent the negative impact of high Se accumulation on the synthesis of key S-metabolites (cysteine, GSH and GLS). Therefore, *D. tenuifolia* appeared to be superior over the other species in this respect. However, because of its high capacity to accumulate Se, plants should be preferentially supplied with Se concentration lower than 5 μM in order to ensure a safer Se intake via consumption of fresh leaves. Otherwise, a greater amount of leaf fresh material (~30 g) from *E. sativa* grown with −10 μM Se could be more safely consumed.

## Figures and Tables

**Table 1:** Effects of selenate treatment on the content of individual glucosinolates identified in *Eruca sativa* and *Diplotaxis tenuifolia* plants cultivated in hydroponics. Data are expressed as mg glucosinolate per 100 mg leaf fresh weight (FW). Different letters along rows indicate significant differences (*n* = 3, ±SD, *p* < 0.05) among treatments. Abbreviation DBM-GLS indicates Dimeric-4-mercaptobutyl glucosinolate.

Glucosinolate	Se (μM)				
	0	5	10	20	40
	*E. Sativa*				
**Glucoraphanin**	62.23 ± 25.04a	94.66 ± 21.98a	76.91 ± 21.61a	26.88 ± 7.71b	38.06 ± 0.15b
**Glucocheirolin**	3.77 ± 2.31a	3.35 ± 2.25a	2.15 ± 0.83a	2.48 ± 1.70a	2.59 ± 1.00a
**Glucoerucin**	13.48 ± 3.77a	14.19 ± 4.76a	10.33 ± 2.15a	5.98 ± 2.47b	8.90 ± 1.59ab
**DMB-GLS**	64.50 ± 7.44a	68.69 ± 6.18a	47.67 ± 7.62b	42.55 ± 3.54b	54.73 ± 0.80b
**Glucosativin**	2.84 ± 1.23ab	2.98 ± 1.34ab	1.93 ± 0.67b	2.17 ± 0.36ab	2.98 ± 0.09a
**Neoglucobrassicin**	1.38 ± 0.37a	1.92 ± 0.88a	1.49 ± 0.29a	0.78 ± 0.24a	2.71 ± 1.57a
	***D. Tenuifolia***				
**Glucoraphanin**	20.75 ± 6.91ab	31.56 ± 13.51a	25.64 ± 9.54ab	8.96 ± 6.98b	12.68 ± 5.22b
**Glucocheirolin**	1.26 ± 0.41a	1.12 ± 0.37a	0.72 ± 0.24a	0.82 ± 0.27a	0.86 ± 0.28a
**Glucoerucin**	4.49 ± 1.49a	4.73 ± 1.57a	3.44 ± 1.14a	1.99 ± 1.66a	2.97 ± 0.98a
**DMB-GLS**	21.50 ± 9.16a	22.89 ± 10.63a	15.89 ± 8.29a	14.18 ± 10.72a	18.24 ± 6.08a
**Glucosativin**	0.95 ± 0.31a	0.99 ± 0.33a	0.64 ± 0.21a	0.72 ± 0.24a	0.99 ± 0.33a
**Neoglucobrassicin**	0.46 ± 0.15a	0.64 ± 0.21a	0.49 ± 0.16ab	0.26 ± 0.08b	0.90 ± 0.30a

**Table 2:** Effects of selenate treatment on the content of free amino acids in leaves of *E. sativa* and *D. tenuifolia* plants cultivated in hydroponics. Data are expressed as mg amino acid per 100 g tissue fresh weight (FW). Different letters along rows indicate significant differences (*n* = 3, ± SD, *p* < 0.05) among treatments.

Amino Acid (mg/100g FW)	Se (μM)				
	0	5	10	20	40
	*E. Sativa*				
**Phenylalanine**	7.29 ± 0.97a	6.15 ± 0.98ab	4.58 ± 0.85b	4.05 ± 1.37b	4.92 ± 1.21b
**Isoleucine**	2.59 ± 0.22a	3.07 ± 0.23a	2.06 ± 0.47a	2.36 ± 0.81a	2.57 ± 0.88a
**Leucine**	0.24 ± 0.13b	0.27 ± 0.09b	0.39 ± 0.13b	0.25 ± 0.11b	0.65 ± 0.18a
**Histidine**	13.56 ± 0.11a	7.63 ± 0.15b	8.92 ± 0.06b	11.78 ± 1.15a	12.63 ± 1.08a
**Tyrosine**	0.91 ± 0.16a	0.59 ± 0.25b	0.50 ± 0.13b	0.52 ± 0.27b	0.56 ± 0.14b
**Tryptophan**	1.25 ± 0.10b	1.13 ± 0.31ab	1.10 ± 0.30ab	1.18 ± 0.30ab	1.56 ± 0.18a
**Arginine**	0.10 ± 0.03a	0.04 ± 0.02b	0.05 ± 0.01b	0.06 ± 0.03ab	0.07 ± 0.01b
**Glutamine**	72.39 ± 6.84a	30.32 ± 7.04b	32.22 ± 12.53b	42.09 ± 15.49b	29.63 ± 2.61b
**Valine**	5.82 ± 0.68a	2.70 ± 0.47c	3.32 ± 0.43bc	3.96 ± 0.68b	4.20 ± 0.91ab
**Proline**	83.08 ± 17.18a	47.56 ± 8.61b	55.44 ± 16.58ab	65.52 ± 15.82ab	56.16 ± 8.44b
**Se-cysteine**	-	0.21 ± 0.11a	0.32 ± 0.23a	0.34 ± 0.92a	0.31 ± 0.06a
	***D. Tenuifolia***				
**Phenylalanine**	1.11 ± 0.21b	0.93 ± 0.20b	1.53 ± 0.69ab	1.19 ± 0.12b	1.88 ± 0.54a
**Isoleucine**	0.73 ± 0.15a	0.33 ± 0.15b	0.36 ± 0.06b	0.41 ± 0.19b	0.58 ± 0.12ab
**Leucine**	0.39 ± 0.24ab	0.36 ± 0.13b	0.32 ± 0.11b	0.49 ± 0.21ab	0.84 ± 0.38a
**Histidine**	4.51 ± 2.20a	3.82 ± 1.14a	1.27 ± 0.27b	1.26 ± 0.48b	1.87 ± 0.80b
**Alanine**	0.87 ± 0.56ab	0.91 ± 0.38ab	1.26 ± 0.49a	0.64 ± 0.18b	0.63 ± 0.07b
**Tyrosine**	0.34 ± 0.09b	0.25 ± 0.09b	0.23 ± 0.05b	0.24 ± 0.11b	0.56 ± 0.03a
**Tryptophan**	0.53 ± 0.20a	0.95 ± 0.18a	0.74 ± 0.38a	1.11 ± 0.53a	0.66 ± 0.10a
**Arginine**	0.18 ± 0.03ab	0.22 ± 0.02a	0.16 ± 0.03b	0.15 ± 0.02b	0.16 ± 0.06ab
**Glutamine**	6.37 ± 0.63b	5.55 ± 0.45b	6.20 ± 1.26b	7.06 ± 0.59ab	8.46 ± 1.25a
**Glutamic acid**	3.60 ± 1.11a	3.82 ± 1.13a	3.36 ± 0.78a	3.97 ± 1.35a	3.56 ± 0.53a
**Valine**	3.71 ± 0.83a	2.86 ± 0.27a	2.89 ± 0.61a	3.46 ± 1.07a	3.00 ± 0.48a
**Lysine**	0.58 ± 0.12a	0.63 ± 0.13a	0.64 ± 0.13a	0.51 ± 0.11a	0.65 ± 0.09a
**Proline**	0.91 ± 0.15b	1.94 ± 0.55b	3.20 ± 0.95b	15.48 ± 10.09a	10.78 ± 8.39ab
**Methionine**	0.21 ± 0.03a	0.18 ± 0.03a	0.18 ± 0.01a	0.20 ± 0.08a	0.21 ± 0.08a
**Se-cysteine**	-	0.69 ± 0.05a	0.87 ± 0.09a	0.76 ± 0.26a	0.73 ± 0.19a

**Table 3:** Effects of selenate treatment on the content of detected polyphenols in leaves of *Eruca sativa* and *D. tenuifolia* plants cultivated in hydroponics. Data are expressed as mg phenol compound per 100 g leaf fresh weight (FW). Different letters along rows indicate significant differences (*n* = 3, ±SD, *p* < 0.05) among treatments. K = Kaempferol. Q = Quercetin. I = Isorhamnetin.

Phenol Compound (mg/100g FW)	Se (μM)				
	0	5	10	20	40
	*E. Sativa*				
**K-3-sinapoil-triglucoside-7-glicoside**	0.46 ± 0.15a	0.20 ± 0.10ab	0.19 ± 0.05b	0.00 ± 0.00d	0.06 ± 0.01c
**K-3-diglucoside-7-glicoside**	0.24 ± 0.07a	0.16 ± 0.09a	0.16 ± 0.06a	0.23 ± 0.07a	0.20 ± 0.07a
**Q-3-glucoside**	0.27 ± 0.04b	0.28 ± 0.03b	0.45 ± 0.04a	0.38 ± 0.03a	0.26 ± 0.04b
**Q-3,4′-diglucoside**	1.49 ± 0.08a	1.30 ± 0.18a	2.88 ± 1.17a	2.41 ± 0.22a	1.24 ± 0.20a
**K-3,4′-diglucoside**	13.07 ± 0.32a	11.94 ± 1.03ab	12.67 ± 1.32ab	12.19 ± 1.05ab	10.38 ± 0.27b
**I-3,4′-diglucoside**	1.25 ± 0.12a	0.83 ± 0.21a	2.08 ± 1.03a	1.54 ±0.39a	0.89 ± 0.19a
**K-3-O-feruloildiglucoside-7-O-glucoside**	0.34 ± 0.06a	0.34 ± 0.06a	0.32 ± 0.12a	0.32 ± 0.08a	0.31 ± 0.06a
**K-3-glucoside**	1.90 ± 0.12a	1.87 ± 0.10a	1.32 ± 0.63ab	1.89 ± 0.20a	0.92 ± 0.08b
**I-3-glucoside**	0.28 ± 0.03a	0.28 ± 0.02a	0.54 ± 0.29a	0.45 ± 0.20a	0.22 ± 0.10a
**Q-3-glucoside 3′ (6-sinapoilglucoside)**	0.44 ± 0.08a	0.42 ± 0.04a	0.22 ± 0.04b	0.00 ± 0.00c	0.20 ± 0.05b
**K-3-(2-sinapoil-glucoside)-4′-glucoside**	2.01 ± 0.08a	2.38 ± 0.15a	0.65 ± 0.10b	0.19 ± 0.10c	0.49 ± 0.12b
**K-3-O-feruloil glucoside-7-O-glucoside**	0.56 ± 0.04a	0.58 ± 0.05a	0.47 ± 0.07a	0.24 ± 0.02b	0.58 ± 0.04a
			***D. Tenuifolia***		
**Q-3-glucoside**	0.27 ± 0.07a	0.20 ± 0.05a	0.14 ± 0.08a	0.22 ± 0.04a	0.21 ± 0.04a
**Q-3,4′-diglucoside**	1.04 ± 0.03a	0.54 ± 0.10b	0.48 ± 0.14b	0.74 ± 0.12b	0.52 ± 0.14b
**Q-3,3′,4′-triglucoside**	1.11 ± 0.04a	0.51 ± 0.13bc	0.46 ± 0.05c	0.73 ± 0.16b	0.48 ± 0.6c
**K-3,4′-diglucoside**	0.71 ± 0.04a	0.30 ± 0.06b	0.41 ± 0.05b	0.37 ± 0.04b	0.34 ± 0.03b
**I-3,4′-diglucoside**	0.60 ± 0.24a	0.44 ± 0.14a	0.35 ± 0.12a	0.56 ± 0.12a	0.33 ± 0.10a
**Q-3,4′-diglucoside 3′ (6-sinapoilglucoside)**	0.48 ± 0.04	0.17 ± 0.04	0.07 ± 0.04	0.39 ± 0.04	0.21 ± 0.04
**Q-3,4′-diglucoside 3′ (6-feruloilglucoside)**	0.16 ± 0.06ab	0.15 ± 0.06ab	0.06 ± 0.04b	0.31 ± 0.12a	0.22 ± 0.10ab
**I 3-glucoside**	0.26 ± 0.05a	0.27 ±0.04a	0.27 ± 0.03a	0.22 ± 0.03a	0.30 ± 0.04a

## Supplementary Materials

The following are available online at https://www.mdpi.com/2223-7747/8/3/68/s1, Figure S1: Growth in agar of *E. sativa* and *D. tenuifolia* in the presence of selenate concentrations ranging within 0–40 µM. On the right, average total Se concentration in the plants; Figure S2: Plants of *E. sativa* and *D. tenuifolia* grown in hydroponics with selenate concentrations ranging within 0–40 µM; Table S1: Fragmentation pattern of glucosinolates identified in leaves of *E. sativa* and *D. tenuifolia* plants., Table S2: Fragmentation pattern of phenolic compounds identified in leaves and roots of *E. sativa* and *D. tenuifolia* plants, Table S3: Sequences of primers used in qRT-PCR reactions.
