# Supplementary material for: Selenium Biofortification Differentially Affects Sulfur Metabolism and Accumulation of Phytochemicals in Two Rocket Species (Eruca Sativa Mill. and Diplotaxis Tenuifolia) Grown in Hydroponics
Source: Plants (Basel). 2019 Mar 16;8(3):68. doi: 10.3390/plants8030068 (PMC6473880; doi:10.3390/plants8030068)
Supplement: Supplementary file 1 [file plants-08-00068-s001.pdf]

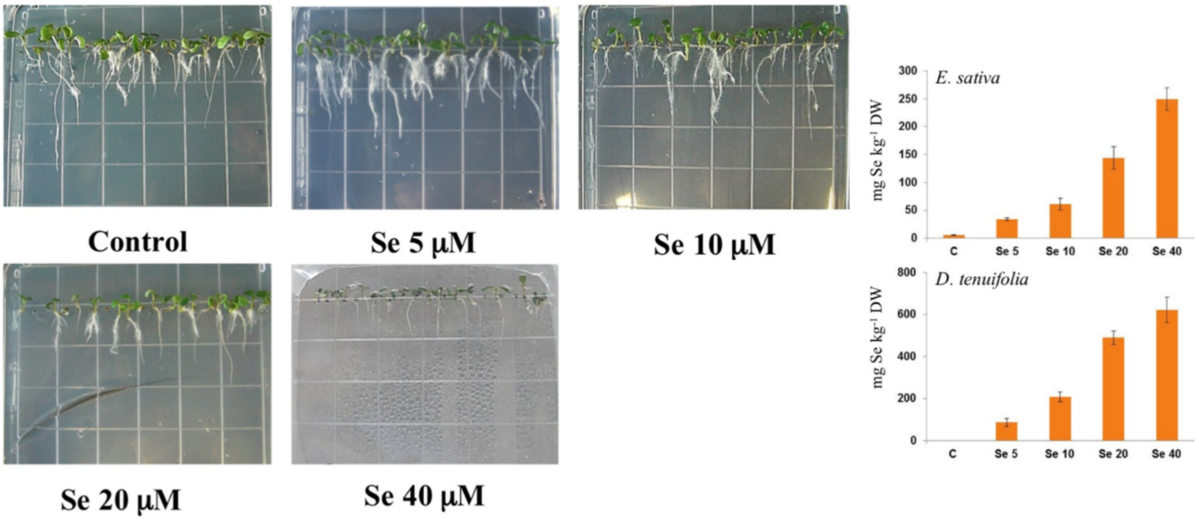

Figure S1. Growth in agar of *E. sativa* and *D. tenuifolia* in the presence of selenite concentrations ranging within 0–40 μM. On the right, average total Se concentration in the plants.

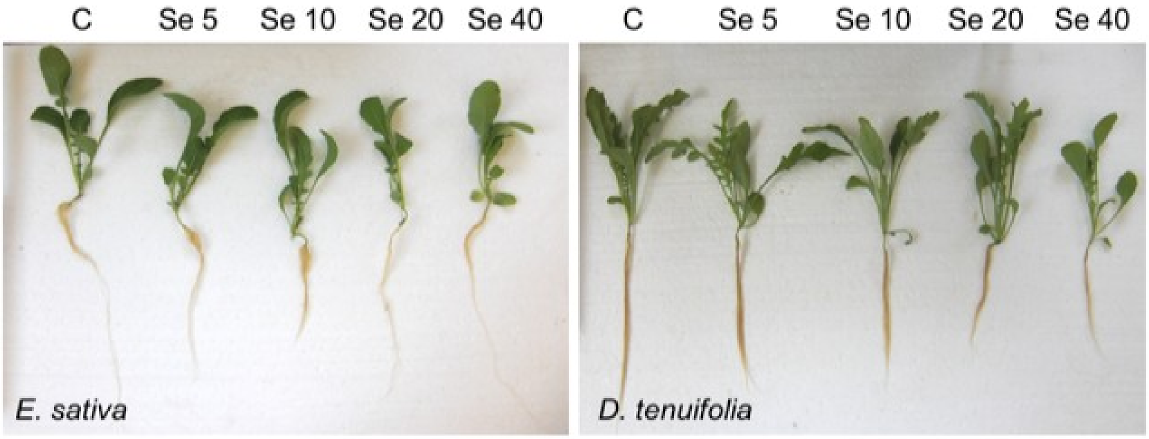

Figure S2. Plants of *E. sativa* and *D. tenuifolia* grown in hydroponics with selenate concentrations ranging within 0–40 μM.

Table 1S. Fragmentation pattern of glucosinolates identified in leaves of *E.sativa* and *D. tenuifolia plants*. Compounds were detected in positive ion mode observing sodium adduct $[M+Na]^+$or potassium adduct $[M+K]^+$ pseudomolecular ions. Abbreviation DBM-GLS indicate Dimeric-4-mercaptobutyl glucosinolate. RT = retention time.

Glucosinolate	Fragmentation	$[M+Na]^+$	$[M+K]^+$	RT(min)
Glucoraphanin	316, 218, 136	380		2,129
Glucocheirolin	198, 164, 146	360		2.511
Glucoerucin	202	364	380	5.442
Glucosativin		328		6.645
Neoglucobrassicin		421		7.01
DMB-GLS	513, 479	675	691	6.645

Table 2S. Fragmentation pattern of phenolic compounds identified in leaves and roots of rocket plants. $[M+H]^+$ - protonated adduct pseudomolecular ions; RT = Retention Time.

Polyphenol	Fragmentation	$[M+H]^+$	RT (min)
K-3-sinapoil-triglucoside-7-glicoside	301, 179	1139	6.7
K-3-diglucoside-7-glicoside	463, 301	771	7.0
Q-3-glucoside	976, 815, 609, 447	463	7.1
Q-3.4′-diglucoside	285, 257, 151	625	7.2
K-3.4′-diglucoside	447, 285, 255, 151	609	7.2
I-3.4′-diglucoside	447, 315, 300, 285	639	7.5
K-3-O-feruloildiglucoside-7-O-glucoside	653, 285	947	9.1
K-3-glucoside	785, 285, 591	447	9.7
I-3-glucoside	314, 285, 271	477	9.8
Q-3-glucoside 3′ (6-sinapoilglucoside)	609, 447	831	10.1
K-3-(2-sinapoil-glucoside)-4′-glucoside	669, 463, 301	815	10.2
K-3-O-feruloil glucoside-7- O-glucoside	623, 447, 285	785	10.5
Q-3.3′.4′-triglucoside	625, 463	787	625, 463
Q-3.4′-diglucoside 3′ (6-sinapoilglucoside)	831, 669, 463	993	477, 315
Q-3.4′-diglucoside 3′ (6-feruloilglucoside)	831	963	831, 669, 463

Table 3S. Sequences of primers used in qRT-PCR reactions.

Gene product name	Forward primer 5′-3′	Reverse Primer 5′-3′
SULTR1;1	TGTTCATCACACCGCTCTTC	TGCTGCGTCAATGTCAATAAG
SULTR1;2	ATGGCTGGATGTCAAACTGC	TCAGAGGAATCACTGCGTTG
SULTR2;1	TTGGGCTACAAGAAACTCGTC	CTGAAAATCCCGAAAGAAGC
ATPS1	CCCTATCCTTTTGCTTCATCC	GTGCTGCTTCATCCTCCAAC
ATPS2	CATCAAGAGGAACA TCATCAGC	TTACAGGCTATCTCCAAAACAGC
ATPS4	TGTTCATCACACCGCTCTTC	TGCTGCGTCAATGTCAATAAG
MYB28	CCCAAGCAGAAAGGTTTCAA	CCCTAAACTTGGGACTAACAACC
BCAT	CCTACTTTGTCCTTCACGC	TCTATGTCTGCTACCTCCG
MAM1	TGCAACCACTGTCAACATCG	GTCAACTTGTCTTGCTCCC
UGT74B1	GATTCCATCGGCTTACCTTG	CCAAACGAACCAAACGAAAC
MYR	GCGAAGAGAACGAACCATTC	GCAACACCGAAGATGAAGTC
ACT1	AGCATGAAGATCAAGGTGGTG	CTGACTCATCGTACTCTCCCT
